# Inability of Physicians and Nurses to Predict Patient Satisfaction in the Emergency Department

**DOI:** 10.5811/westjem.2015.9.28177

**Published:** 2015-12-14

**Authors:** Matthew C. DeLaney, David B. Page, Ethan B. Kunstadt, Matt Ragan, Joel Rodgers, Henry E. Wang

**Affiliations:** University of Alabama School of Medicine, Department of Emergency Medicine, Birmingham, Alabama

## Abstract

**Introduction:**

Patient satisfaction is a commonly assessed dimension of emergency department (ED) care quality. The ability of ED clinicians to estimate patient satisfaction is unknown. We sought to evaluate the ability of emergency medicine resident physicians and nurses to predict patient-reported satisfaction with physician and nursing care, pain levels, and understanding of discharge instructions.

**Methods:**

We studied a convenience sample of 100 patients treated at an urban academic ED. Patients rated satisfaction with nursing care, physician care, pain level at time of disposition and understanding of discharge instructions. Resident physicians and nurses estimated responses for each patient. We compared patient, physician and nursing responses using Cohen’s kappa, weighting the estimates to account for the ordinal responses.

**Results:**

Overall, patients had a high degree of satisfaction with care provided by the nurses and physicians, although this was underestimated by providers. There was poor agreement between physician estimation of patient satisfaction (weighted κ=0.23, standard error: 0.078) and nursing estimates of patient satisfaction (weighted κ=0.11, standard error: 0.043); physician estimation of patient pain (weighted κ=0.43, standard error: 0.082) and nursing estimates (weighted κ=0.39, standard error: 0.081); physician estimates of patient comprehension of discharge instruction (weighted κ=0.19, standard error: 0.082) and nursing estimates (weighted κ=0.13, standard error: 0.078). Providers underestimated pain and patient comprehension of discharge instructions.

**Conclusion:**

ED providers were not able to predict patient satisfaction with nurse or physician care, pain level, or understanding of discharge instructions.

## INTRODUCTION

Patient satisfaction is an increasingly important metric that is being measured in emergency departments (ED) across the country. Patient satisfaction scores have a wide effect on outcomes for patients, providers, and healthcare organizations. For patients, previous studies have associated high levels of patient satisfaction with improved outcomes.^1^ In terms of outcomes for providers, high satisfaction scores have been associated with a lower rate of patient complaints and possibly a lower rate of malpractice claims.^2^,^3^ In addition, patient satisfaction scores are commonly tied to physician compensation. A recent study reported that 59% of physicians reported that their compensation was tied to their satisfaction scores in what is essentially a pay-for-performance model. Conversely 20% of providers reported that their employment had been threatened as a result of patient satisfaction data.^4^ For healthcare organizations, patient satisfaction scores are playing an increasing role in determining overall reimbursement. In 2012 the Center for Medicare and Medicaid services (CMS) began development of a Hospital Consumer Assessment of Healthcare Providers and Systems (HCAPS) patient satisfaction survey. While the exact implementation of this program is unclear, it stands to tie a significant portion of reimbursement from Medicaid to the results of the patient satisfaction survey.^5^

Given the increasing importance placed on patient satisfaction in EDs nationwide, extensive efforts have been made to identify factors that contribute to patient satisfaction; and interventions have been developed to improve overall satisfaction. Staff interpersonal skills, perceived wait times, effective and timely analgesia, the use of ED structure information cards and follow-up telephone calls have all been shown to influence patient satisfaction.^6^–^10^ Various strategies have been employed to improve overall patient satisfaction from adjusting provider staffing to standardizing communication with patients, and even playing background music in the ED.^11^–^13^

To date there is minimal evidence to suggest that providers are able to accurately predict patient satisfaction. As an increasing number of programs are designed to improve patient satisfaction, it is crucial that providers are able to accurately estimate patient satisfaction. Our study evaluated the provider’s ability to predict patient satisfaction.

## METHODS

### Study Design and Setting

This cross-sectional was performed at the University of Alabama at Birmingham (UAB) Hospital, an urban academic teaching hospital. The ED has 50 beds, all of which are private, and sees approximately 72,000 patients per year. The institutional review board at UAB approved this study.

#### Selection of Participants

Based on research staff availability, a convenience sample of 100 eligible patients was selected at the time of discharge from the ED. Patients were enrolled over a two-month period (July–August 2013), seven days a week between 6am and 11pm. Research staff used the Cerner FirstNet Triage and Tracking system to identify patients being dispositioned. Eligibility criteria included English-speaking ambulatory patients over 18 years old and deemed healthy enough to participate in the study. Exclusion criteria included intoxicated patients, prison inmates, patients with a primary psychiatric diagnosis, patients 18 years of age and under, and patients who entered the department as a trauma alert.

#### Method of Measurement

At the time of disposition, research assistants approached eligible patients in their private treatment rooms. Prior to the patient exiting the department, study staff administered a face-to-face interview that consisted of 10 questions regarding their satisfaction with the visit and their pain management. Each of the questions were Likert items that allowed the patient to rate their satisfaction with physician care, nursing care, pain level and their understanding of the discharge instructions on a scale of 1 to 5.

Our survey was designed to resemble the format of our Press Ganey ED patient satisfaction surveys. Responses were measured on a scale of 1 to 5.^14^ Our survey asked the patient to verbally rate their satisfaction with their nursing care, satisfaction with their physician, their pain level at the time of discharge and their understanding of discharge instructions. (See Addendum 1.) After interviewing the patient, research assistants interviewed the treating resident physician and nurse. Physicians and nursing staff were blinded to patient responses.

#### Data Analysis

We used descriptive statistics to characterize the patient sample. We used kappa statistics to evaluate the agreement between patient and physician responses and patient and nurse responses on like questions from their respective interviews. To account for the ordered data, we used weighted kappa. To provide a clear analysis, we simplified the five-point scale used in the interview into a three-point scale. On the three-point scale, we categorized scores of 1–2 as “satisfied,” 3 as “neither” satisfied nor dissatisfied and 4–5 as “dissatisfied.”

We used Excel to manage data (Microsoft, Inc., Redmond, Washington) and performed statistical analyses with Stata v. 13.0 (Stata, Inc., College Station, Texas).

## RESULTS

### Demographics (Table 1)

The mean age of patients completing the survey was 49.9 years. Thirty-one patients were rated with an emergency severity index of 2, 61 were rated 3, and 8 were rated 4. Sixty-six patients spent less than four hours in the ED, and a total of 45 patients were admitted.

### Patient Satisfaction (Tables 2 and 3)

Overall patients had a high degree of satisfaction with 99% of patients reporting that they were satisfied with the nursing care and 96% reporting a similar degree of satisfaction with physician care. Despite the generally high level of satisfaction, there was poor agreement between the patient’s responses and the provider’s estimation of these responses. There was poor agreement between nurse and patient responses (weighted κ=0.11, standard error: 0.043);); similarly, there was poor agreement between physician and patient responses (weighted κ=0.23, standard error: 0.078). Providers tended to underestimate the patient’s degree of satisfaction. Providers underestimated patient satisfaction with nursing in 12% of cases and with physicians in 18% of cases. There were no cases where nurses overestimated patient satisfaction and only one case where physicians overestimated patient satisfaction.

### Pain Levels (Table 4)

Patients reported a wide variety of pain levels at discharge with ~62% reporting no pain, 20% reporting moderate pain, and 18% reporting that they were experiencing the a high level of pain. Both physician and patient responses (weighted κ=0.43, standard error: 0.082) and nurse and patient responses (weighted κ=0.39, standard error: 0.081) had poor agreement when estimating a pain level. Both nurses and physicians underestimated the patient’s pain in 20% of cases. In 8% of the nurse’s cases and 6% of the physician’s cases, the patient reported severe pain while the providers predicted that the patient was in no pain.

### Discharge Instructions (Table 5)

Patients reported that they fully understood their discharge instructions in 87% of the cases. Nurses underestimated patient comprehension in 6% of cases and physicians underestimated this response in 17% of cases. Nurses overestimated comprehension in 12% of cases, compared to 10% with physicians. There was poor agreement between physician and patient responses (weighted κ=0.19, standard error: 0.082) as well as nurse and patient responses. (weighted κ=0.13, standard error: 0.078)

We found that there was poor agreement between patient responses and provider estimates of the patient responses across all aspects of our survey. Overall providers tended to underestimate the level of patient satisfaction. In addition, providers underestimated patient’s level of pain at discharge, and tended to underestimate a patient’s comprehension of their discharge instructions.

## DISCUSSION

In our study we found that providers are not able to reliably predict patient responses to questions similar to those commonly found on patient satisfaction surveys. While previous studies have attempted to identify factors that may contribute to patient satisfaction, few studies to date have evaluated provider’s ability to predict patient satisfaction.

Boudreaux et al. examined whether providers were able to accurately estimate patient satisfaction. Providers were asked to predict the patient’s response to a 22-question survey that focused on overall satisfaction and included questions regarding satisfaction with nursing and physician care and understanding of discharge instructions. Responses were obtained from 478 patients and 59 providers. The authors found that providers consistently underestimated the patients’ reported satisfaction.^15^

Our study addressed a major limitation that was found in the paper by Boudreaux et al. Rather than asking providers to estimate patient satisfaction with a particular visit, they were asked to estimate the overall survey results for all the patients that had been seen in the department during the study period. Asking providers to provide such a general estimate of satisfaction provides little information in terms of their ability to predict patient responses in particular situations. Our study asked providers to predict a response for an individual patient interaction. By focusing on a particular patient interaction, our data more closely evaluated the provider’s ability to assess various variables and predict patient satisfaction.

In our study we found that providers have difficulty predicting patient satisfaction. Our providers tended to underestimate the level of satisfaction that patients had with their care. Due to a high overall rate of satisfaction, our providers did not encounter a large number of unsatisfied patients. Providers did correctly identify all patients that were either dissatisfied or neither satisfied nor dissatisfied their care. Unfortunately, based on the extremely low incidence of dissatisfaction and relatively small sample size, our study does not provide compelling evidence that providers can accurately predict dissatisfied patients.

We found that providers underestimated patient pain at the time of discharge. Cases where a provider underestimates the patient’s pain accounted for 20% of our visits. These visits may represent instances where the patients received inadequate analgesia. Previous studies have suggested that adequate pain control can improve patient satisfaction.^16^ Our data suggests that providers are not able to reliably predict a patient degree of pain at discharge. Given the association between patient satisfaction and pain level, ED providers should focus on performing a more accurate assessment of a patient’s pain and providing appropriate analgesia prior to discharge.

The majority of our patients reported that they understood their discharge instructions. Despite the reportedly high level of comprehension, providers underestimated patient comprehension in 10–12% of cases. Boudreaux et al. found that patient comprehension of discharge instructions was a significant predictor of overall patient satisfaction.^17^ Providers should continue to focus on identifying patients who have poor comprehension of discharge instructions in order to improve overall satisfaction.

Our study illustrates the difficulty providers have when they are asked to predict patient responses to questions regarding patient satisfaction. Nationwide as more attention is placed on improving overall patient satisfaction, various initiatives have been developed in an effort to enhance the patient’s ED visit. Programs such as hourly rounding on ED patients, developing a system for follow-up communication after discharge from the ED have been credited with improving patient satisfaction scores. Typically these initiatives are applied to broad range of patients, such as all patients discharged home, rather than targeting specific patients who are at risk of having a low level of patient satisfaction.^18^

The inability of providers to accurately predict patient responses may lead to poor resource utilization when implementing programs to improve patient satisfaction. We found that providers had difficulty distinguishing between satisfied and unsatisfied patients. Identifying unsatisfied patients could allow departments to focus resources on improving particular at-risk interactions rather than applying broad initiatives to all patients in the ED. Our study demonstrated that providers have difficulty predicting patient responses to a wide variety of satisfaction metrics. As efforts to improve patient satisfaction continue to grow, departments should also focus on enhancing a provider’s ability to accurately assess patient satisfaction.

## LIMITATIONS

We surveyed patients at the end of their ED visit, while previously most patients have received surveys at home several days to weeks after their ED visit. This delay between discharge and responding to the survey may influence the patient’s responses; therefore, our results may not be generalizable.

We relied on patient assessment of comprehension of their discharge instructions. We chose this subjective estimation to approximate the questions found on common patient satisfaction surveys. It is possible that patients overestimated their actual comprehension, as previous studies have reported a much lower rate of comprehension than we found, with Engel et al. reporting significant knowledge deficits in terms of return instructions and home care instructions in up to 80% of patients discharged from the ED.^19^ While our data accurately reflects patient self-assessment, it may not reflect actual comprehension of discharge instructions.

We used a five-point pain scale to assess patient’s satisfaction with their pain control, as is common in other satisfaction surveys. Commonly in the ED pain is scored on a 0–10 point scale. There may be some inconsistency in patient responses when they are asked to respond using a five-point scale after using a 10-point scale during their ED visit. While patient responses using a five-point scale may not be directly transferrable to the standard 10-point system, our data accurately reflect the scoring used on common patient satisfaction studies.

A disproportionately large subset of included patients were admitted, potentially skewing results.

## CONCLUSION

Physicians and nurses are not able to accurately predict patient responses to standard patient satisfaction surveys. As increasing emphasis is being placed on patient satisfaction nationwide, efforts should be made to improve a provider’s ability to predict a patient’s level of satisfaction with his or her care.

## Figures and Tables

**Table 1:** Demographics of patients included in patient satisfaction study.

Demographics	Demographic values (SD)
Mean age (SD)	49.9 (17.3)
Emergency severity index (ESI) (Number of patients)	
2	31
3	61
4	8
Mean ESI (SD)	2.77 (0.58)
Mean emergency department length of stay (SD)	3.66 hours (2.33)
Admission rate	45%

*ESI,* emergency severity index

**Table 2:** Comparison between nurse assessment and patient report of satisfaction with emergency department nursing care. Percentages reflect column percentages. Weighted kappa for agreement between nurse and patient ratings=0.11 (Standard Error: 0.043).

	Patient reported satisfaction with nurse		
	
Nurse estimation of satisfaction	Satisfied	Neither	Dissatisfied
Satisfied	87 (87.9)	0 (0)	0 (0)
Neither	10 (10.1)	1 (100)	0 (0)
Dissatisfied	2 (2)	0 (0)	0 (0)

**Table 3:** Comparison between physician assessment and patient report of satisfaction with emergency department physician care. Percentages reflect column percentages. Weighted kappa for agreement between physician and patient ratings=0.20 (Standard error: 0.058).

	Patient reported satisfaction with physician		
	
Physician estimation of satisfaction	Satisfied	Neither	Dissatisfied
Satisfied	78 (81.3)	1 (33.3)	0 (0)
Neither	13 (13.6)	1 (33.3)	0 (0)
Dissatisfied	5 (5.2)	1 (33.3)	1 (100)

**Table 4:** Comparison between nurse and physician assessment and patient report of pain level. Percentages reflect column percentages. Weighted kappa for agreement between nurse and patient ratings=0.39 (Standard error: 0.081). Weighted kappa for agreement between physician and patient ratings=0.43 (Standard error: 0.082).

	Patient reported pain level		
	
Pain level estimation by provider	No pain	Moderate pain	Severe pain
Nurse estimation of pain level			
No pain	53 (85.5)	6 (30)	8 (44.5)
Moderate pain	7 (11.3)	10 (50)	6 (33.3)
Severe pain	2 (3.2)	4 (20)	4 (22.2)
Physician estimation of pain level			
No pain	52 (83.9)	10 (50)	6 (33.3)
Moderate pain	8 (12.9)	7 (35)	4 (22.2)
Severe pain	2 (3.2)	3 (15)	8 (44.5)

**Table 5:** Comparison between nurse and physician assessment and patient report of how well the patient understood his/her discharge instructions. Percentages reflect column percentages. Weighted kappa for agreement between nurse and patient ratings=0.13 (Standard error: 0.078). Weighted kappa for agreement between physician and patient ratings=0.19 (Standard error: 0.082).

	Patient reported understanding of discharge instructions		
	
Estimation of understanding of discharge intructions by provider	Fully understand	Somewhat understand	Don’t understand
Nurse estimation of understanding of discharge instructions			
Fully understand	81 (93.1)	6 (85.7)	4 (66.7)
Somewhat understand	5 (5.8)	1 (14.3)	2 (33.3)
Don’t understand	1 (1.1)	0 (0)	0 (0)
Physician-estimation of understanding of discharge instructions			
Fully understand	70 (80.5)	6 (85.7)	2 (33.3)
Somewhat understand	14 (16.1)	1 (14.3)	2 (33.3)
Don’t understand	3 (3.4)	0 (0)	2 (33.3)
